# Structure Elucidation and Computationally Guided Synthesis of SSZ‐43: A One‐Dimensional 12‐Ring Zeolite with Unique Sinusoidal Channels

**DOI:** 10.1002/anie.202115087

**Published:** 2022-02-16

**Authors:** Maria Roslova, Viktor J. Cybulskis, Mark E. Davis, Stacey I. Zones, Xiaodong Zou, Dan Xie

**Affiliations:** ^1^ Department of Materials and Environmental Chemistry Stockholm University 106 91 Stockholm Sweden; ^2^ Chemical Engineering California Institute of Technology Pasadena CA 91125 USA; ^3^ Chevron Technical Center 100 Chevron Way Richmond CA 94801 USA

**Keywords:** Heterogeneous Catalysis, Molecular Modeling, Structure Determination, Targeted Synthesis, Zeolites

## Abstract

The structure of zeolite SSZ‐43 was determined by 3D electron diffraction, synchrotron X‐ray powder diffraction, and high‐resolution transmission electron microscopy. The SSZ‐43 framework forms one‐dimensional, sinusoidal 12‐ring channels from 5^4^6^1^ *butterfly units* commonly found in other zeolites, but with unique 6.5×6.5 Å apertures and 12‐ring 6.5×8.9 Å windows perpendicular to the channels. SSZ‐43 crystals are intergrowths of two polytypes: *≈*90 % orthorhombic polytype A with ABAB stacking of the 12‐rings, and ≈10 % monoclinic polytype B with ABCABC stacking. Molecular modeling performed on the idealized Si‐SSZ‐43 structure along with empirical relationships for zeolite selectivity in boron‐ and aluminum‐containing synthesis gels were used in a combined approach to design new di‐quaternary ammonium organic structure‐directing agents (OSDAs). Experimental trials demonstrated that the new OSDAs produced SSZ‐43 over a broader range of compositions than previous mono‐quaternary OSDAs.

## Graphical abstract

After two decades since its discovery, the structure of zeolite SSZ‐43 has been determined as a novel framework comprised of a primitive orthorhombic crystal system with monoclinic faulting that forms unique, 1D 12‐ring sinusoidal channels. A combination of molecular modeling and empirical relationships for zeolite selectivity was used to design two new di‐quaternary OSDAs and produce SSZ‐43 across a wide range of high SiO_2_ compositions.

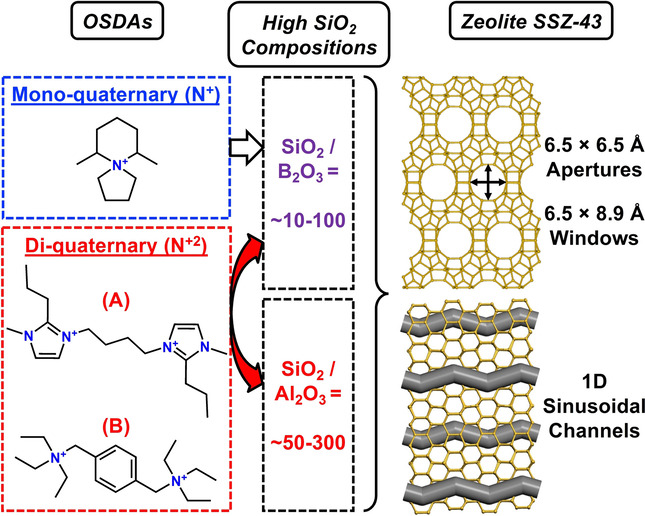

## Introduction

**Figure 1:**
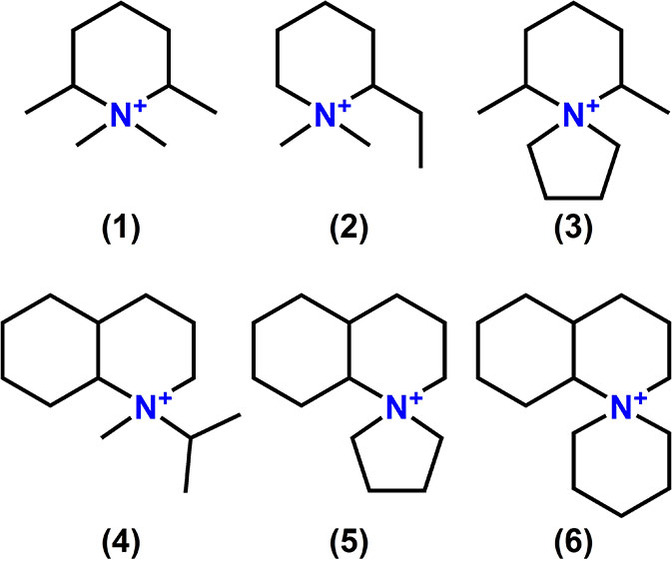
Previously reported piperidinium‐ and decahydroquinolinium‐derived mono‐quaternary ammonium hydroxide OSDAs for the synthesis of SSZ‐43.^[11]^

Zeolites are widely used in catalytic, adsorption, and separation processes.[^1^, ^2^, ^3^, ^4^, ^5^, ^6^, ^7^] These important industrial applications continue to drive the development of zeolites across broader composition spaces (e.g., silica‐to‐heteroatom ratios) and promote the discovery of entirely new structures. The synthesis of novel zeolites has largely been enabled by using amine or quaternary ammonium molecule as the organic structure‐directing agent (OSDA), where the size and shape of the OSDA often geometrically align with the confining voids of the target zeolite host. Obtaining detailed structural information of zeolites is a prerequisite for understanding their catalytic, adsorptive, and other physicochemical properties. Furthermore, once the zeolite structure is known, more feasible and comprehensive synthesis routes can be devised, often with guidance from computational tools. For example, SSZ‐52 (i.e., **S**tandard Oil **S**ynthetic **Z**eolite‐52), a small‐pore, cage‐containing, aluminosilicate zeolite with 8‐ring windows was discovered by Lee and Zones at Chevron in 2001,^[8]^ but little additional development occurred until its structure (assigned code **SFW** by the Structure Commission of the International Zeolite Association) was determined in 2013.^[9]^ The structure solution of SSZ‐52 revealed its close tectonic relationship to SSZ‐13, a small‐pore, aluminosilicate zeolite of **CHA** topology, and signaled its potential use as an emissions abatement catalyst. Initially, commercial scale‐up of SSZ‐52 was hindered by the complexity of the original OSDA, but computational modeling efforts later identified alternative organo‐cation guest molecules to permit a more economical synthesis route.^[10]^ Similar to the case of SSZ‐52, zeolite SSZ‐43 was synthesized in 1999 by Lee et al.^[11]^ from a series of piperidinium‐ and decahydroquinolinium‐derived mono‐quaternary ammonium compounds as OSDAs (Figure 1). A comparison of previously reported adsorption results for SSZ‐43[^12^, ^13^] along with those for a series of known zeolite frameworks suggests that SSZ‐43 may contain 12‐ring pores and possess additional structural similarities to large‐pore zeolites SSZ‐48 (**SFE**) and SSZ‐31 (*STO). However, since its discovery over two decades ago, the structure of SSZ‐43 remained unsolved.

Developing new or modified medium‐ (10‐ring) and large‐pore (12‐ring) zeolites is often key to enabling significant advances in refining and petrochemistry because the confining void spaces and pore architectures of these frameworks are often well suited for interactions with small hydrocarbon molecules in fuels, lubricants, and petrochemicals. Thus, there is considerable interest in understanding the structural details of novel zeolites, such as SSZ‐43. However, as is often the case with synthetic zeolites, samples of SSZ‐43 are polycrystalline and prohibit structural solution by established single‐crystal X‐ray diffraction techniques. In addition, SSZ‐43 is a partially disordered material, making structural derivation challenging from powder X‐ray diffraction (PXRD) patterns alone. Fortunately, recent developments in three‐dimensional electron diffraction (3D ED) in combination with high‐resolution transmission electron microscopy (HRTEM)[^14^, ^15^, ^16^] have facilitated the structure determination of many complex zeolites.[^17^, ^18^, ^19^, ^20^, ^21^, ^22^, ^23^, ^24^, ^25^, ^26^, ^27^, ^28^] Here, by using these emerging characterization methods, we have unraveled the structure of SSZ‐43 along with finer details of stacking faults. The SSZ‐43 framework contains a unidirectional 12‐ring channel that traverses the *c*‐axis in a sinusoidal manner, making this structure most unusual among other known 1D, large‐pore zeolites. Additionally, we report a new computationally guided synthesis route for SSZ‐43 that was developed by combining molecular modeling of the idealized SSZ‐43 framework structure with experimentally derived relationships in zeolite phase selectivity for boron‐ and aluminum‐containing synthesis gels. This approach was used to design two different di‐quaternary ammonium OSDAs, which were identified according to their stabilization energies in the idealized framework structure and further validated through laboratory experiments. Using the newly discovered OSDAs, both boron‐ and aluminum‐containing, high‐silica (i.e., SiO_2_/M_*x*_O_*y*_>12) SSZ‐43 zeolites could be synthesized in hydroxide (OH^−^) media.

## Results and Discussion

**Figure 2:**
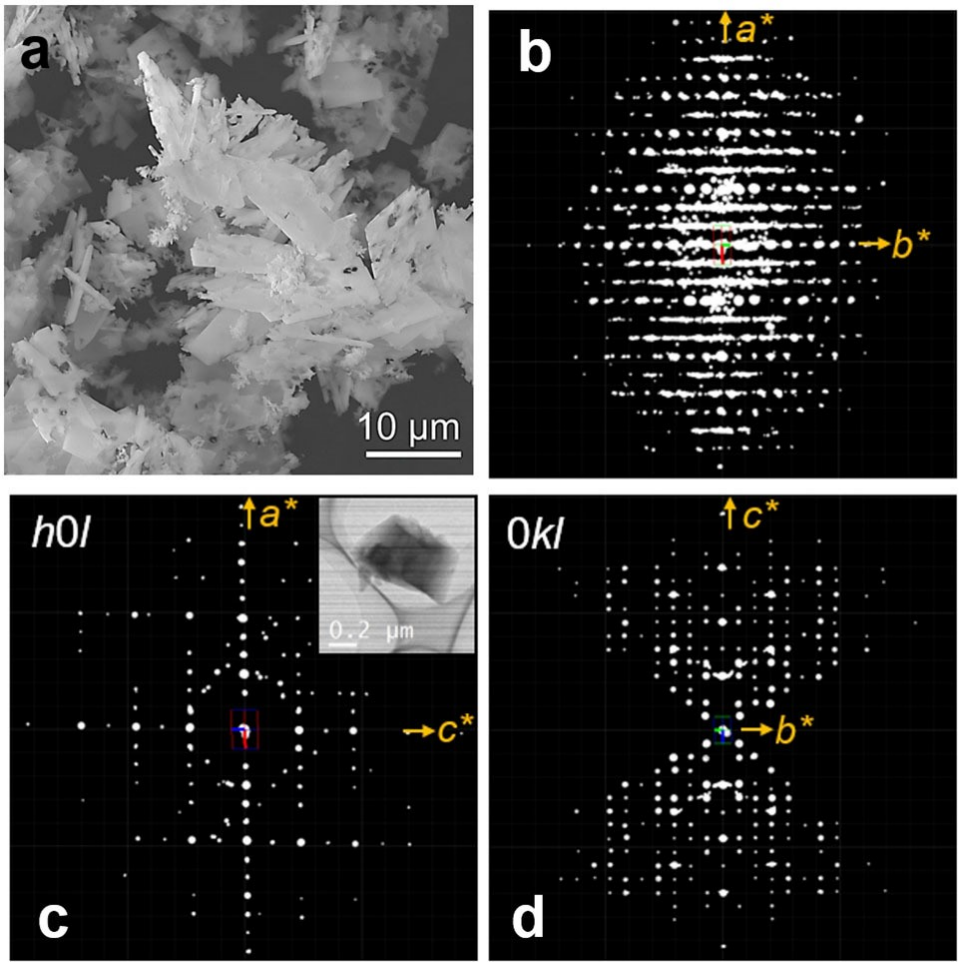
a) SEM image of B‐SSZ‐43 synthesized from OSDA Molecule 3 in Figure 1 showing the flake‐like morphology of the crystallites. b)–d) 3D ED pattern of SSZ‐43. The reconstructed 3D reciprocal lattice showing b) the diffuse scattering, c) the 2D *h*0 *l*, and d) the 0*kl* 2D slices, as visualized by REDp software.^[16]^ The inset in (c) shows the corresponding crystal image.

The SEM image of the as‐made B‐SSZ‐43 synthesized from OSDA Molecule 3[^29^, ^30^] (Figure 1 and Supporting Information Section 1.1) shows the flake‐like morphology of the individual crystallites (≈4–10 μm, Figure 2a). Attempts to solve the structure by using PXRD data or even index the XRD pattern were unsuccessful due to the severe peak overlapping, even in the case of high‐resolution PXRD synchrotron data. Thus, the structure of B‐SSZ‐43 was investigated by continuous rotation electron diffraction (cRED), which is the latest protocol of 3D ED methods. cRED data were collected from multiple individual crystals on a JEOL JEM‐2100 microscope with a LaB_6_ cathode operating at 200 kV using *Instamatic* software.^[31]^ Additional characterization details, including PXRD and N_2_ adsorption data for the calcined B‐SSZ‐43 sample (OSDA Molecule 3), are provided in Section 2 and Figures S1 and S2 of the Supporting Information. From 3D reciprocal space reconstructed from the cRED data using REDp software,^[14]^ diffuse streaks were observed (Figure 2b–d). The cRED data could be indexed using two distinct types of unit cells. We found most SSZ‐43 crystallites can be indexed with a primitive orthorhombic unit cell of *a=*12.51, *b=*29.03, *c=*17.30 Å. From the 2D slices, the reflection conditions can be deduced as the following: *h*0*l*: *l*=2*n*; 0*kl*: *k*=2*n*, which gives two possible space groups *Pbc*2_1_ (No. 29) and *Pbcm* (No. 57). The highest centrosymmetric space group *Pbcm* was chosen for structure determination. Datasets from seven different crystals were merged to increase the data completeness and redundancy (Table 2). Additionally, several datasets of SSZ‐43 could be indexed with a monoclinic unit cell of *a=*12.61, *b=*17.31, *c=*14.29 Å, *β*=106.4° and space group *P*2_1_/*m*. Both the orthorhombic and monoclinic structures of SSZ‐43 could be solved and refined from the cRED data. Details of crystallographic data and the structure refinements are given in Table 1 and Supporting Information Section 2.

**Table 1:** Selected crystallographic data for SSZ‐43. The cell parameters marked with an asterisk were obtained from synchrotron PXRD data, not cRED data.

Parameter	SSZ‐43 Ortho	SSZ‐43 Mono
Datasets merged	7	1
Space group	*Pbcm* (57)	*P*2_1_/*m* (11)
Averaged unit cell parameters		
*a* [Å]	12.324(2)*	12.61(3)
*b* [Å]	28.435(1)*	17.31(2)
*c* [Å]	16.898(1)*	14.29(2)
*β* [°]	90	106.4(1)
Total No. of reflections	99 086	5065
No. of unique reflections	6701	2820
No. of reflections with *F*_o_>4*σ*(*F*_o_ *I*)	3786	1879
*R*_int_	0.3082	0.2282
*CC*_1/2_	99.8	99.2
Completeness [%]	99.5	89
Resolution cut‐off [Å]	1.00	1.00
No. parameters	392	156
No. restrains	192	332
*R*_1_ (*I*>2*σ*(*I*))	0.2793	0.3163
*R*_1_ (all data)	0.3128	0.3322
GOF	1.690	2.006

Initially, the average structure of SSZ‐43 was solved in the orthorhombic space group *Pbcm* (57) by using SHELXT^[32]^ in Olex2 software^[33]^ from the dataset merged from seven crystals selected by hierarchical cluster analysis using *Edtool*.^[16]^ A check for higher symmetry by the ADSYMM routine in PLATON^[34]^ confirmed the *Pbcm* space group. The structure was further refined by SHELXL^[35]^ using atomic structure factors for electrons.^[36]^ The orthorhombic and monoclinic structures of SSZ‐43 are denoted polytype A and B, respectively. Their atomic coordinates are given in Tables S1 and S2, respectively.

**Figure 3:**
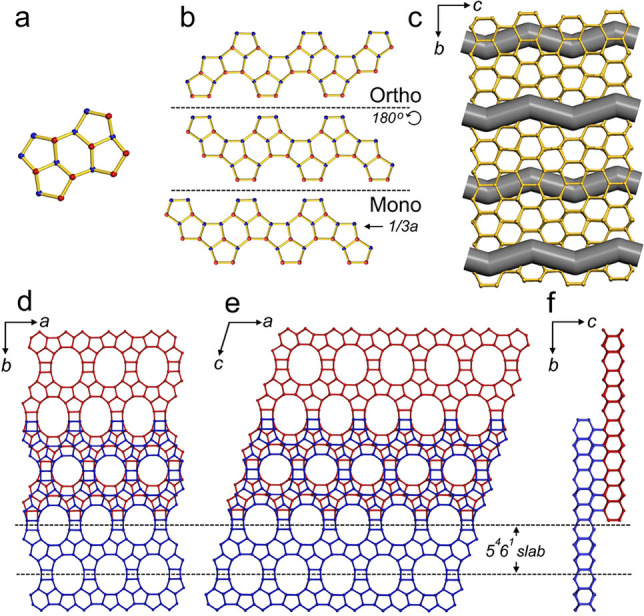
Assembly of the SSZ‐43 framework structure. a) A *butterfly unit* of SSZ‐43 with up (U) and down (D) configurations of TO_4_ tetrahedra are shown in red and blue, respectively. b) Neighbouring 5^4^6^1^ slabs are rotated by 180° around the *a*‐axis in orthorhombic polytype A and shifted by 1/3*a* to each other in monoclinic polytype B. c) Sinusoidal channels in the SSZ‐43 structure. d) Orthorhombic polytype A and e) monoclinic polytype B SSZ‐43 framework. f) Side‐view showing connectivity between top and bottom layers in polytype A. Top layer is blue, bottom layer is red. Bridging O atoms have been omitted for clarity.

The framework structures of both polytype A and B of SSZ‐43 contain 14 unique T‐atoms (T=tetrahedral) and can be assembled by using the 5^4^6^1^ *butterfly units* connected via 4‐rings similar to *STO (e.g., SSZ‐31). The *butterfly unit* can be constructed using two *mel* [4^1^5^2^6^2^] composite building units (CBUs), one *jbw* [6^4^], and two incomplete cages. As shown in Figure 3, extension of the *butterfly units* along the *a*‐axis forms the SSZ‐43 5^4^6^1^ slab. The neighboring 5^4^6^1^ slabs are rotated by 180° around the *a*‐axis in the orthorhombic polytype A and shifted by 1/3*a* in the monoclinic polytype B, and further connected to form layers containing 12‐rings (Figure 3b). The TO_4_ tetrahedra in each 12‐ring are arranged either up (U) or down (D) in a UDUUDUDUDDUD configuration. The layers are further connected to the next sheet with the same configuration via 6‐rings. Thus, one unit cell of SSZ‐43 contains two 6‐ring “honeycomb” blocks that are either shifted by ±1/2*b* relative to one another in the orthorhombic structure, or by ±*c* in the monoclinic structure as shown in Figure 3d, e.

The orthorhombic SSZ‐43 framework hosts sinusoidal channels with 6.5×6.5 Å apertures along the *c*‐axis, which are arranged in an ABAB stacking sequence along the *b*‐axis (Figure 3d). The channels are formed by 12‐ring windows having open diameters of 6.5×8.9 Å. The monoclinic SSZ‐43 framework contains the same neighboring 5^4^6^1^ slabs as the orthorhombic one, but they are shifted by ±1/3*a* relative to one another, rather than flipped, leading to an ABCABC stacking sequence (Figure 3e). The diffuse streaks observed along the b*‐axis for reflections with *h*≠3*n* (Figure 2b) indicate the presence of structural disorder in the SSZ‐43 framework. Such streaks likely originate from the stacking disorder along the *b*‐axis caused by the irregular shift of the 12‐ring channels by ±1/3*a* (i.e., mixture of the ABAB stacking and ABCABC stacking). This disorder results in the appearance of sharp spots for reflections with *h*=3*n* and streaks for reflections with *h*≠3*n*.

**Figure 4:**
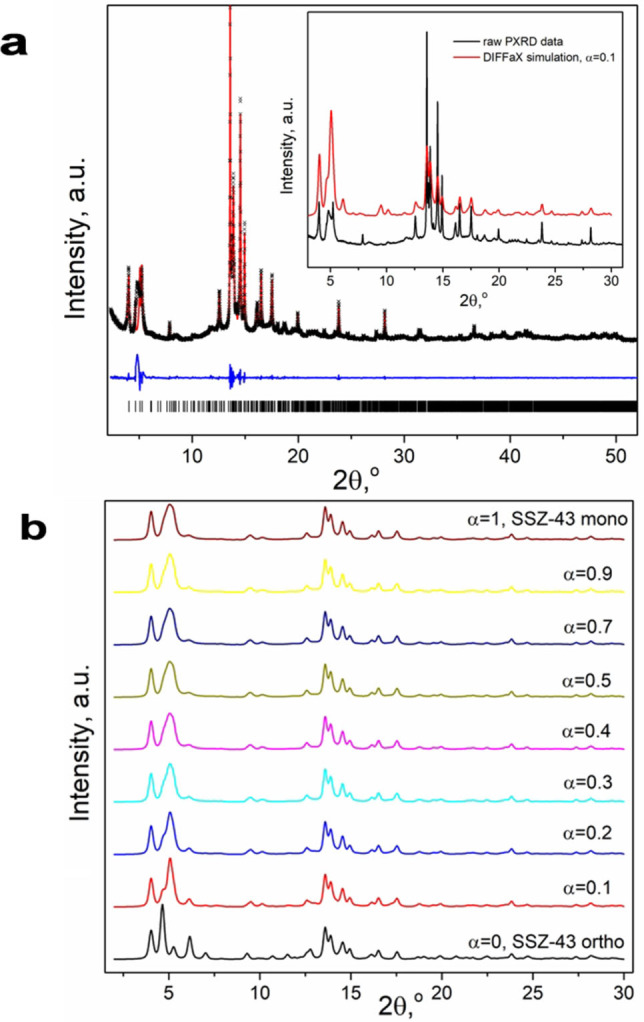
a) Profile fitting of synchrotron PXRD pattern for SSZ‐43 (black=experimental, red=calculated, blue=difference). Refined peak positions indicated by the black ticks are shown in the bottom. Inset shows comparison of synchrotron PXRD pattern (bottom) and DIFFaX simulated PXRD pattern assuming 10 % monoclinic faulting in orthorhombic SSZ‐43 polytype A (top). b) PXRD patterns simulated by DIFFaX for different fault probabilities.

To better understand the nature of stacking faults in the SSZ‐43 structure, synchrotron PXRD data (*λ*=0.99995 Å) was used for further analysis. The orthorhombic unit cell parameters of *a*=12.324(2), *b*=28.435(1), *c*=16.898(1) Å as derived from profile fitting, were used to refine the structural model obtained from cRED data, with restraints applied to all Si−O distances (1.61 Å) and O−Si−O angles (109°). The refined atomic coordinates were then used to generate the base layer for DIFFaX simulation^[37]^ (Supporting Information Section 2). By reasoning that the fault direction in the DIFFaX simulation should be fixed along the vertical *b*‐axis for the original orthorhombic unit cell, the layer in parallel to the original *ac* plane was set as the base layer and the stacking vector was set as 1/2*b*. Then, 1/3*a* and 2/3*a* shifts were applied to generate the second and the third layers, respectively. To ensure that the desired layer stacking sequence was produced, the DIFFaX‐simulated PXRD patterns for the structurally ordered end‐members, i.e., orthorhombic polytype A (ABAB‐stacking) and monoclinic polytype B (ABCABC‐stacking), were verified by comparison to the calculated PXRD patterns from the refined ED structural models as shown in Figure 4b. The faulting probability α=0 corresponds to the orthorhombic polytype A, whereas α=1 corresponds to the monoclinic polytype B. After validating these two end‐members, DIFFaX was used to simulate faulting across the full range of stacking probabilities (α=0.0–1.0) as shown in Figure 4b. As the extent of monoclinic faulting increases, the peak associated with the (100) reflection at 2 *θ*=4.65° (*d*=12.32 Å) shifts to higher angles and merges with that of the (110) reflection at 2 *θ*=5.07° (*d*=11.31 Å) to form a broad peak at ≈5° when α≥0.2. Comparison of the synchrotron PXRD pattern for SSZ‐43 with the simulated data (Figure 4a inset) reveals that SSZ‐43 contains mainly orthorhombic polytype A with ≈10 % monoclinic polytype B (α=0.1). The peak intensity discrepancies between the experimental PXRD data and DIFFaX simulation shown in Figure 4a are likely due to the presence of OSDAs, boron heteroatoms and the preferred orientation introduced by the flake‐like morphology of the SSZ‐43 crystallites, neither of which can be simulated by DIFFaX.

**Figure 5:**
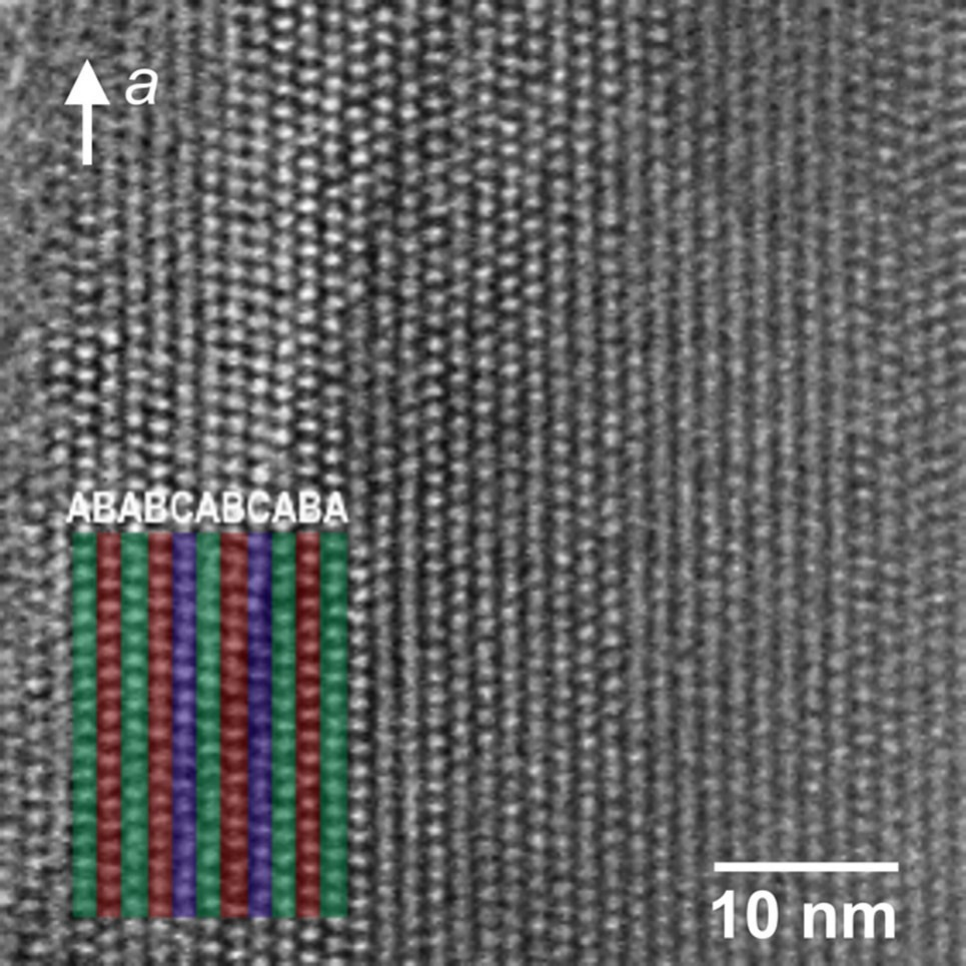
HRTEM image of the 12‐ring channel stacking sequence in a faulted SSZ‐43 domain.

HRTEM real space imaging was performed on a series of different SSZ‐43 crystals to directly observe the local stacking faults in SSZ‐43 and confirm layer shifts in the *a*‐axis direction as shown in Figure 5. Here, the local stacking (A, B, or C) in the thin area of an SSZ‐43 crystal specimen was analyzed and color‐coded. Stacking sequences of ABABCABCAB were present, but no reiteration of A, B, or C layers was observed, nor could any clear long‐range stacking sequence ordering be found.

After the structural models of the SSZ‐43 polytype A and B were derived, a molecular modeling study on OSDA‐zeolite interaction was performed to design new, energetically favorable OSDAs for this framework, and to computationally guide the experimental synthesis of borosilicate and aluminosilicate SSZ‐43 by using these identified molecules (Supporting Information Section 3). We note that pure SiO_2_ framework compositions were used because the precise crystallographic locations of heteroatoms (e.g., B, Al) within SSZ‐43 and the other zeolite structures considered in this study are not known. Additionally, we limited the modeling to van del Waals (vdW) interaction energies between OSDA molecules and the target zeolite framework without including other inorganic cations (e.g., Na^+^, K^+^) or H_2_O molecules in the calculation. First, we determined the optimized vdW interaction energies between the six previously known OSDAs for SSZ‐43^[11]^ (Figure 1) and the idealized all‐silica SSZ‐43 framework (i.e., orthorhombic) according to the method described in Section 3 of the Supporting Information. For each mono‐quaternary organo‐cation (Molecules 1–6), the minimum vdW interaction energy—calculated as the energy difference between the zeolite framework with OSDA occluded, the isolated OSDA, and the empty SSZ‐43 unit cell—occurred at a loading of *n*=4 OSDA molecules per unit cell. The optimal OSDA loadings agreed with experimental results obtained by thermogravimetric analysis (TGA). As shown in Table 2, the vdW interaction energies between Molecules 1–6 and SSZ‐43 ranged from −5.2 to −6.7 kJ (mole Si)^−1^ with the decahydroquinolinium‐derived organo‐cations (Molecules 4–6) being most favorable.

**Table 2:** Calculated vdW interaction energies for OSDA Molecules 1–12 in the framework structures of SSZ‐43, SSZ‐31 (*STO polymorph I), and SSZ‐35 (**STF**) along with experimentally observed products for a variety of synthesis gel compositions.[^11^, ^13^, ^38^, ^39^]

OSDA Molecule	Modeling Predictions			Experimental Results		
	OSDA‐Zeolite vdW Interaction Energy [kJ (mole Si)^−1^]			SiO_2_/Al_2_O_3_		SiO_2_/B_2_O_3_
	SSZ‐43	SSZ‐31 (*STO poly‐I)	SSZ‐35 (**STF**)	50–300	>300	10–100
1	−5.2	−5.1	−9.6	Amorphous	SSZ‐31	SSZ‐35, SSZ‐43
2	−5.2	−5.2	−9.5	SSZ‐31	SSZ‐31	SSZ‐43
3	−5.6	−5.6	−10.9	SSZ‐35	SSZ‐31	SSZ‐35, SSZ‐43
4	−5.7	−5.0	−10.0	Amorphous	Amorphous	SSZ‐43
5	−6.4	−5.9	−9.5	SSZ‐31, SSZ‐43	SSZ‐31, SSZ‐43	SSZ‐43
6	−6.7	−5.4	−6.2	SSZ‐31, SSZ‐43	SSZ‐31, SSZ‐43	SSZ‐43
7	−4.4	−5.4	−8.7	SSZ‐31	SSZ‐31	Amorphous
8	−6.2	−5.8	−12.8	SSZ‐35	SSZ‐35	SSZ‐35
9	−5.7	−4.9	−11.2	SSZ‐35	SSZ‐35	SSZ‐35
10	−5.3	−5.2	−10.4	SSZ‐35	Amorphous	SSZ‐35
11	−5.5	−5.5	−11.1	Amorphous	SSZ‐31	SSZ‐35
12	−5.7	−5.4	−11.0	SSZ‐31, SSZ‐35	SSZ‐31	SSZ‐35

SSZ‐31 (*STO, polymorph I), a one‐dimensional large‐pore zeolite, and SSZ‐35 (**STF**), a one‐dimensional medium‐pore zeolite with large internal cavities, are two zeolites that also crystallize from gel compositions and synthesis conditions similar to those used for SSZ‐43. From a structural standpoint, both SSZ‐43 and *STO contain the same *mel* [4^1^5^2^6^2^] composite building units, these units form the 5^4^6^1^ *butterfly units* that are further connected to themselves to assemble the same building layers. The vdW interaction energies between OSDA Molecules 1–6 (Figure 1) and competing phases along with six additional OSDAs (Molecules 7–12 in Figure S3) that are known to promote the formation of the competing *STO and **STF** phases, but not SSZ‐43,[^11^, ^13^, ^38^, ^39^] were also calculated and included in Table 2 for comparison.

*Intra‐zeolite* comparisons of interaction energies between various OSDAs and a given zeolite structure were used to assess the relative fit between the guest molecule and framework host and guide the experimental synthesis of the zeolite.[^10^, ^40^, ^41^, ^42^] The worse the interaction energy that an OSDA molecule offers, the less likely the OSDA molecule will crystalize the particular zeolite experimentally. Here, the results of experimental syntheses with OSDA Molecules 1–12 for a range of SiO_2_/Al_2_O_3_ (SAR) and SiO_2_/B_2_O_3_ (SBR) in Table 2 indicate that zeolite phase selectivity correlates, in general, with the vdW interaction energy between the guest molecule and framework host for a given zeolite. On the other hand, it is important to note that *inter‐zeolite* comparisons of interaction energies for a given OSDA are not straightforward since the zeolite host framework density (FD) and the percentage of framework atoms available to participate in vdW interactions could vary between different zeolite frameworks.[^43^, ^44^] As a result, vdW interaction energy normalized either by the number of T atoms or by the number of OSDA molecules per unit cell is not ideal, especially when evaluating a cage‐based lower FD phase versus a channel‐based higher FD phase. For example, SSZ‐35 (**STF**) has the lowest FD (16.9 T×10^−3^ Å^−3^) among the three zeolites considered (SSZ‐31, SSZ‐35, SSZ‐43). Furthermore, SSZ‐35 (**STF**) is also the only example here where all framework atoms are exposed to the one‐dimensional channel to interact with the occluded OSDA molecules. Thus, as shown in Table 2, the calculated interaction energies (normalized per each framework Si atom) for Molecules 1–12 are generally lower in SSZ‐35 than in SSZ‐31 and SSZ‐43.

As indicated by the modeling predictions and experimental results in Table 2, the probability of forming a specific zeolite from a given OSDA can also be affected by how well the molecule interacts with other species in the synthesis gel, particularly in the presence of heteroatoms such as B or Al, to influence the kinetics of nucleation and growth for other competing phases. If based solely on ranking the vdW interaction energies for the idealized pure SiO_2_ SSZ‐43 structure or *STO, then the modeling results in Table 2 would indicate that Molecule 8 should very likely promote the formation of either SSZ‐43 or SSZ‐31. Of the twelve OSDAs, the interaction energy of Molecule 8 in SSZ‐43 is the third most favorable for that framework and the second most favorable for *STO. However, neither of these structures could be produced experimentally with Molecule 8, because this OSDA is much more energetically favorable in **STF** so that SSZ‐35 could be formed under a broader range of synthesis conditions and gel compositions.

Often a minor modification to the OSDA can significantly impact the vdW interaction energy between the guest molecule and framework host, resulting in phase selectivity changes for the crystalline products. For example, OSDA Molecules 5 and 7 (Figures 1 and S3, respectively) are similar except that the addition of a methyl group to the azaspiro ring on the latter increases the vdW interaction energy by ≈2 kJ (mole Si)^−1^ and prevents the crystallization of SSZ‐43 across a broad range of synthesis gel compositions. However, in the case of SSZ‐31, the ≈0.5 kJ (mole Si)^−1^ interaction energy increase between Molecules 5 and 7 is not significant enough to affect the product phase selectivity. These results demonstrate that both OSDA‐zeolite stabilization energies and synthesis gel chemistry must be considered simultaneously when designing new molecules or identifying conditions to steer selectivity toward a zeolite of interest while preventing the formation of more thermodynamically stable, undesired structures.

Among the three zeolites (SSZ‐31, SSZ‐35 and SSZ‐43), SSZ‐31 prefers to crystallize from high Si/heteroatom gels, while SSZ‐35 is generally favored at lower Si/heteroatom ratios. Thus, if OSDAs with more favorable vdW interaction energies in SSZ‐43 can be developed, then the experimental conditions to synthesize SSZ‐43 may be expanded to improve its selectivity across broader ranges of gel compositions and avoid formation of the competing *STO and **STF** phases. While molecular modeling predictions and experimentally observed product phase selectivity do not universally agree, the strategy that we have proposed can be useful in guiding the synthesis of desired zeolite structures from similar gel compositions and inorganic conditions that are known to produce competing phases.

To demonstrate this predictive capability for SSZ‐43, we initiated a search for new OSDAs by screening an internal library of existing quaternary ammonium compounds that have been used successfully to synthesize zeolites and remain stable under the harsh conditions (e.g., pH>10, *T*>120 °C, autogenous pressures) during hydrothermal synthesis. Organo‐cations with two charge centers (i.e., di‐quaternary OSDAs) were preferentially screened in an attempt to steer the crystallization products toward SSZ‐43 and away from *STO and **STF**. Since the periodicity of SSZ‐43 along the 12‐ring pore (*c*‐axis) is ≈16.9 Å for the orthorhombic polytype A, or approximately twice the length as monoclinic *STO (≈8.4 Å, *b*‐axis) and **STF** (≈7.5 Å, *c*‐axis), we hypothesized that longer chain di‐quaternary OSDAs could more effectively stabilize SSZ‐43 by providing a better match of size and shape between the guest molecule and host zeolite. For each simulation, the minimum vdW interaction energy between the di‐quaternary OSDA and the idealized all‐silica SSZ‐43 structure was calculated by using the molecular modeling procedure described in Section 3 of the Supporting Information, and then used to rank the OSDA candidates from most‐to‐least energetically favorable. Based on the combined modeling predictions and experimental results for Molecules 1–6 in Table 2 capable of producing SSZ‐43, OSDA candidates with vdW interaction energies above −5.2 kJ (mole Si)^−1^ (i.e., the least favorable vdW interaction energy among Molecules 1–6) were excluded from the ranking. Next, the vdW interaction energies of these remaining di‐quaternary OSDA candidates in the all‐silica **STF** and *STO frameworks were computed. As previously discussed, the interaction energies for Molecules 1–12 (Table 2) in all‐silica **STF** from modeling predictions were, in general, much lower than in the idealized siliceous *STO (poly‐I) and SSZ‐43 structures. So here we elected, as a starting point for the screening process, to exclude di‐quaternary OSDAs with *E*_**STF**_<2×*E*_SSZ‐43_ and E_*STO_<*E*_SSZ‐43_ from consideration.

**Figure 6:**
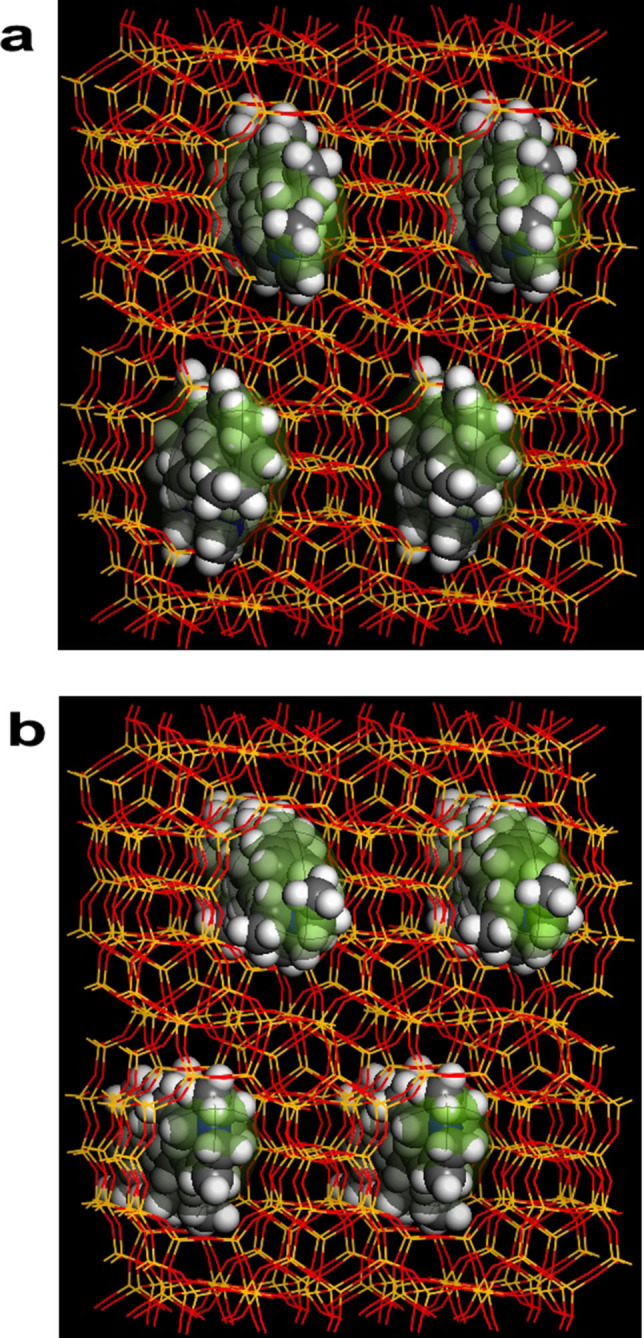
Predicted energy‐minimized configurations of a) Molecule A and b) Molecule B in the 12‐ring sinusoidal channels of SSZ‐43.

Of the original ≈300 candidate OSDAs from the internal library, five di‐quaternary molecules were selected for experimental trials to synthesize B‐SSZ‐43 and Al‐SSZ‐43 according to the method described in Section 1.2 of the Supporting Information. Out of these five molecules, two OSDAs (denoted Molecule A and Molecule B) successfully produced pure SSZ‐43^[45]^ in both aluminosilicate and borosilicate forms ranging from SAR=50–300 and SBR=10–100, respectively, as shown in Table 3. The vdW interaction energies between Molecules A & B and pure SiO_2_ SSZ‐43 were calculated to be −5.8 and −5.4 kJ (mole Si)^−1^, respectively, with optimal loadings of *n*=2 OSDAs per unit cell for both OSDA‐zeolite systems. The lowest energy configurations of Molecules A and B inside the one‐dimensional sinusoidal channels of SSZ‐43 as predicted by molecular modeling are shown in Figure 6a and b, respectively.

**Table 3:** Calculated vdW interaction energies for new di‐quaternary OSDA Molecules A and B in the framework structures of SSZ‐43, SSZ‐31 (*STO polymorph I), and SSZ‐35 (**STF**) along with experimentally observed products for a variety of synthesis gel compositions.

OSDA Molecule	Modeling Predictions			Experimental Results		
	OSDA‐Zeolite vdW Interaction Energy [kJ (mole Si)^−1^]			SiO_2_/Al_2_O_3_		SiO_2_/B_2_O_3_
	SSZ‐43	SSZ‐31 (*STO poly‐I)	SSZ‐35 (**STF**)	50–300	>300	10–100
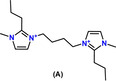	−5.8	−5.6	−9.7	SSZ‐43	SSZ‐31, SSZ‐43	SSZ‐35, SSZ‐43
						
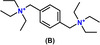	−5.4	−5.1	−9.4	SSZ‐43	Amorphous	SSZ‐43

SEM images along with the PXRD pattern of the as‐made Al‐SSZ‐43 sample synthesized from the new di‐quaternary OSDA Molecule A (Figure S4) indicate that the crystallites exhibit morphological and structural features similar to those in the B‐SSZ‐43 sample synthesized from the original mono‐quaternary OSDA Molecule 3 (Figure 1a). TGA results from the as‐made Al‐SSZ‐43 sample with Molecule A showed ≈9.7 wt% loss in organic mass, consistent with the theoretical loading of two OSDA molecules per unit cell (≈8.3 wt%). Additionally, CHN analysis confirmed that the carbon‐to‐nitrogen ratio in this as‐made sample was 4.1, which agrees with the theoretical value of 4.5 for Molecule A (C_18_H_32_N_4_^2+^).

While Molecule A successfully produced SSZ‐43, formation of competing SSZ‐31 (*STO) and SSZ‐35 (**STF**) phases could not be avoided in either very high SAR (>300) or low SBR (<40) gels. For example, SSZ‐31 (*STO) crystallized in the presence of Molecule A from pure silica (SAR=∞) gel, but SSZ‐43 formed as Al(OH)_3_ was added to the synthesis gel (SAR<500). By contrast, SSZ‐43 was the dominant product in the borosilicate system with gel compositions of SBR>40, but at higher B concentrations (SBR ≈10–40) product selectivity shifted toward SSZ‐35 (**STF**). Similarly, Molecule B also produced B‐SSZ‐43, but required the use of seed crystals in the aluminosilicate gel to crystallize Al‐SSZ‐43 and eliminate the formation of ZSM‐12 (**MTW**) as a frequently observed by‐product. Interestingly, the interaction energy of Molecule 3, the original mono‐quaternary OSDA, was predicted to be ≈0.2 kJ (mole Si)^−1^ more favorable in SSZ‐43 than Molecule B (Tables 2 and 3), yet Molecule 3 was unable to produce Al‐SSZ‐43 even during seed‐assisted syntheses. These results demonstrate that considering only thermodynamic interaction energies between the OSDA and target pure SiO_2_ zeolite may not always be sufficient for devising new synthesis routes, particularly when additional chemical (e.g., heteroatoms, inorganic cations, seeds) and physical (e.g., temperature, time, agitation) factors affect the kinetics of zeolite nucleation and growth. In such cases, empirical relationships for zeolite phase selectivity are useful supplements to computationally guided OSDA design.

## Conclusion

Following its initial discovery over two decades ago, the novel structure of zeolite SSZ‐43 has been determined by using a combination of 3D ED, HRTEM, and synchrotron PXRD structural characterization techniques in conjunction with the faulting simulation program DIFFaX. Two SSZ‐43 polytypes were found, an orthorhombic polytype A with ABAB stacking of the 12‐rings and monoclinic polytype B with ABCABC stacking. The SSZ‐43 crystals contain mainly the orthorhombic polytype A that is interrupted by monoclinic polytype B. Synchrotron PXRD analysis indicates that the SSZ‐43 sample comprises approximately 90 % of the polytype A. The framework structure of SSZ‐43 is unique. It contains one‐dimensional 12‐ring sinusoidal channels (6.5×6.5 Å pore, 6.5×8.9 Å window) constructed from connecting 5^4^6^1^ *butterfly units*. Such atypical features may potentially exhibit useful shape‐selective catalytic properties in applications such as hydrocracking and hydroisomerization of linear alkanes, where isomer product distributions can be influenced significantly by using zeolites with appropriately sized pore openings and surrounding void environments.

An ongoing chemical challenge for synthesizing SSZ‐43 as an aluminosilicate in thermally stable high SAR compositions that are relevant for catalysis, adsorption, and separations is preventing the formation of competing phases, such as SSZ‐31 (*STO) and SSZ‐35 (**STF**). By using the idealized structural model of SSZ‐43, molecular modeling studies were performed to screen and identify thermodynamically favorable OSDA candidates based on the minimum vdW interaction energy between the guest molecule and host framework. Experimental trials showed that two predicted new di‐quaternary OSDAs could selectively produce SSZ‐43 across a broader range of compositions (SAR=50–300+, SBR=10–100) than the previously known mono‐quaternary compounds. The strategy reported here demonstrates that consideration of both OSDA‐zeolite stabilization energies and gel chemistry are needed for rational design of new structure‐directing molecules to steer zeolite phase selectivity in systems that are known to produce multiple competing zeolite structures under similar synthesis conditions.

## Conflict of interest

The authors declare no conflict of interest.

1

## Supporting information

As a service to our authors and readers, this journal provides supporting information supplied by the authors. Such materials are peer reviewed and may be re‐organized for online delivery, but are not copy‐edited or typeset. Technical support issues arising from supporting information (other than missing files) should be addressed to the authors.

Supporting Information

## Data Availability

The data that support the findings of this study are available from the corresponding author upon reasonable request.
