# Supplementary material for: Structure Elucidation and Computationally Guided Synthesis of SSZ‐43: A One‐Dimensional 12‐Ring Zeolite with Unique Sinusoidal Channels
Source: Angew Chem Int Ed Engl. 2022 Feb 16;61(14):e202115087. doi: 10.1002/anie.202115087 (PMC9306729; doi:10.1002/anie.202115087)
Supplement: Supplementary file 1 — Supporting Information [file ANIE-61-0-s001.pdf]

Angewandte Chemie
Eine Zeitschrift der Gesellschaft Deutscher Chemiker

Supporting Information

Structure Elucidation and Computationally Guided Synthesis of SSZ-43: A One-Dimensional 12-Ring Zeolite with Unique Sinusoidal Channels

M. Roslova, V. J. Cybulskis, M. E. Davis, S. I. Zones, X. Zou, D. Xie**

Supporting Information

Table of Contents

1. Synthesis Procedures 2
2. Materials Characterization 3
3. Molecular Modeling 4
4. Tables
   Table S.1. Atomic coordinates of the orthorhombic SSZ-43 structure 5
   Table S.2. Atomic coordinates of the monoclinic SSZ-43 structure 6
5. Figures
   Figure S.1. PXRD patterns for B-SSZ-43 prepared from Molecule 3 7
   Figure S.2. N_2 adsorption for B-SSZ-43 prepared from Molecule 3 7
   Figure S.3. OSDAs known to form competing ***STO** and **STF** phases 8
   Figure S.4. Al-SSZ-43 zeolite synthesized from OSDA Molecule A 8
6. Accession Codes 8
7. References 9

1. Synthesis Procedures

1.1 Synthesis of B-SSZ-43 from Mono-quaternary OSDA for Structure Solution

OSDA Synthesis. *(6R,10S)-2,6-dimethyl-1-azaspiro[4.5]decan-5-ium hydroxide* (Figure 1, Molecule 3) was prepared according to the method described by Shvets et al [1] as shown below in Scheme 1. Approximately 5.7 g of $NaOH_{(s)}$ (J.T. Baker, >98%) and 30.6 g of 1,4-dibromobutane (Sigma Aldrich, 99%) were added to 140 ml of distilled H_2O in a 500-ml round-bottom flask and stirred vigorously (~1,000 rpm) under reflux (100 °C). Next, 16.1 g of *cis*-2,6-dimethylpiperidine (TCI, ≥99.0%) was added dropwise to the mixture over a period of ~30 min under reflux. The mixture was allowed to react under reflux while stirring for 12 hours overnight, and then quenched in a dry ice bath. Approximately 70 ml of chilled 50 wt.% $NaOH_{(aq)}$ solution was added to the mixture under intense stirring (~1,000 rpm) followed by slow addition of ~20 g of $NaOH_{(s)}$. The mixture was further cooled with dry ice and allowed to stir for 2 hours, which led to the formation of a crystalline product and oil phase.

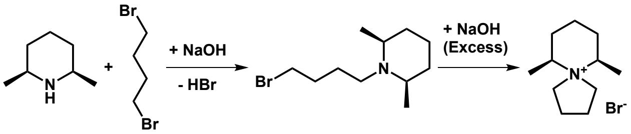

Scheme 1. Synthesis of (6*R*,10*S*)-2,6-dimethyl-1-azaspiro[4.5]decan-5-ium bromide. [1]

The oil was separated mechanically, the solids were filtered off, and the products were extracted from the aqueous phase with 5, 100-ml aliquots of chloroform. The organic phase was dried with anhydrous $MgSO_4$, filtered, evaporated under vacuum, washed with diethyl ether, and filtered again to recover the (6*R*,10*S*)-2,6-dimethyl-1-azaspiro[4.5]decan-5-ium bromide. The OSDA structure and purity were verified by ^{1}H and ^{13}C NMR in [D_6]-dimethyl sulfoxide performed on a Bruker 400 MHz spectrometer. The bromide form of the OSDA was converted to the hydroxide form by ion exchange with Dowex Monosphere 550A resin in distilled H_2O, and then titrated with 0.01 N HCl on a Mettler-Toledo DL22 autotitrator.

B-SSZ-43 Synthesis. The synthesis was performed according to a modified procedure described by Lee et al.[2] with calcined boron beta zeolite as the sole source of silicon and boron. Boron beta zeolite was hydrothermally synthesized from Cab-O-Sil M-5 (Cabot, 99.8%), H_3BO_3 (J.T. Baker, 99.5%), tetraethylammonium hydroxide (Sigma Aldrich, 20 wt.%), and distilled H_2O in the absence of alkali metal cations according to the method described by Senapati et al.[3] The B-SSZ-43 synthesis gel of molar composition 1.0 SiO_2 : 0.25 R^+ : 0.03 B_2O_3 : 0.05 Na^+ : 0.30 OH^- : 33.1 H_2O, where R is the (6*R*,10*S*)-2,6-dimethyl-1-azaspiro[4.5]decan-5-ium hydroxide OSDA, was prepared by adding 0.5 g of calcined boron beta zeolite, 0.45 g of $NaOH_{(aq)}$ (Sigma Aldrich, 1.0 N), and 0.11 g of distilled H_2O into 4.83 g of the OSDA solution (0.432 mmol OH^- g^{-1}). The mixture was stirred (~150 rpm) at room temperature for 2 hours. After addition of 0.01 g of B-SSZ-43 seed crystals to the solution gel, the mixture was transferred to a 23-ml PTFE-lined stainless-steel autoclave (Parr Instrument Co.) and placed inside of a stationary convection oven at 150 °C. Aliquots of the reaction media were collected at 15, 21, 28, and 40 days to monitor the hydrothermal conversion of calcined boron beta to B-SSZ-43. The hydrothermal synthesis was terminated after 55 days under static conditions at 150 °C when no further changes in the crystal structure and morphology were observed by powder X-ray diffraction (PXRD) and scanning electron microscopy (SEM). The solid products were separated from the supernatant by centrifugation (4,500 rpm, 10 min), and then washed with acetone and distilled H_2O in alternating wash/centrifuge/decant cycles (2 washes with each solvent, ~15 ml solvent per

wash). The solids were dried in air at 100 °C for 24 hours, and then post-synthetically treated with 0.1 N KOH solution (500 ml KOH $(g_{solid})^{-1}$) while stirring (~500 rpm) under reflux (100 °C) for 24 hours to dissolve the un-reacted boron beta. Subsequent centrifugation and washing with distilled H_2O resulted in the pure B-SSZ-43 product (~34% yield) consisting of plate-like crystals (~4-10 μm).

1.2 Synthesis of Al- and B-SSZ-43 from New, Di-quaternary OSDAs Identified by Molecular Modeling

OSDA Synthesis. *3,3'-(butane-1,4-diyl)bis(1-methyl-2-propyl-1H-imidazol-3-ium) iodide* (Table 3, Molecule A*) and N,N'-(1,4-phenylenebis(methylene))bis(N,N-diethylethanaminium) bromide* (Table 3, Molecule B) were purchased from MolPort Inc. The di-quaternary salt was dissolved in deionized water (1 ml H_2O $(mmol_{salt})^{-1}$), and then BIO-RAD AG 1-X8 hydroxide-based ion exchange resin was added (1.1 g_{resin} $(mmol_{salt})^{-1}$) to the solution. The resulting slurry was left to stir gently overnight. The slurry was filtered, and the filtrate was analyzed by titration with a 0.1 N HCl standard solution. The exchange afforded the dihydroxide derivative in over 90% yield. Generally, SSZ-43 was synthesized [4] according to the method described in the examples below by heating a mixture of the OSDA, a SiO_2 source, NaOH, an aluminum or a boron source, and H_2O. The molar ratios of a representative synthesis gel are: SiO_2 : 0.020-0.002 T_2O_3 (T = B or Al) : 0.05-0.10 NaOH : 0.08-0.10 $R(OH)_2$: 35 H_2O, where R is the di-quaternary OSDA.

Al-SSZ-43 Synthesis. In a typical synthesis, 2.53 g of deionized H_2O, 0.11 g of 50 wt.% $NaOH_{(aq)}$, 4.47 g of 10.19 wt.% Molecule A hydroxide solution, 0.014 g of 50 wt.% Al_2O_3 dry gel (Barcroft™ 0250 aluminum hydroxide, SPI Pharma), and 3.00 g of LUDOX© AS-30 colloidal silica were mixed in a PTFE liner. The resulting gel was stirred until it became homogeneous. The PTFE liner was then capped and sealed within a stainless-steel autoclave (Parr Instrument Co.). The autoclave was then placed in an oven and heated at 170 °C for 11 days while continuosly rotating at ~43 rpm. The solid products were recovered from the cooled autoclave by centrifugation, washed with deionized H_2O, and dried in air at 95 °C. The resulting product was analyzed by PXRD and SEM and shown to be pure Al-SSZ-43. The product had a SiO_2/Al_2O_3 molar ratio (SAR) of ~142, as determined by inductively coupled plasma (ICP) elemental analysis (performed by Galbraith Laboratories at Knoxville, TN).

B-SSZ-43 Synthesis. In a typical synthesis, 9.91 g of deionized H_2O, 0.19 g of 50 wt.% $NaOH_{(aq)}$, 21.78 g of 6.05 wt.% Molecule B hydroxide solution, and 3.00 g of calcined boron beta zeolite (Si/B = 50) were mixed in a PTFE liner. The resulting gel was stirred until it became homogeneous. The PTFE liner was then capped and sealed within a stainless-steel autoclave (Parr Instrument Co.). The autoclave was then placed in an oven and heated at 150 °C for 7 days under static conditions. The solid products were recovered from the cooled autoclave by centrifugation, washed with deionized H_2O, and dried at 95 °C. The resulting product was analyzed by PXRD and SEM and shown to be pure B-SSZ-43. The product had a SiO_2/B_2O_3 molar ratio (SBR) of ~72, as determined by ICP elemental analysis (performed by Galbraith Laboratories at Knoxville, TN).

2. Materials Characterization

Powder X-ray diffraction (PXRD). Preliminary PXRD patterns were recorded on a Panalytical Empyrean instrument with Cu Kα radiation. Synchrotron PXRD data were collected on the 2-1 Powder Diffraction beamline at the Stanford Synchrotron Radiation Light Source (SSRL). Data were collected at ambient temperature from 2.3° to 61.2° with a

step size of 0.007° 2θ using a wavelength of 0.99995 Å. Jana2006 software [5] was used for LeBail fit of PXRD data.

Scanning Electron Microscopy (SEM). The SEM images were collected on a JEOL JSM-7000F microscope equipped with a field-emission gun. An acceleration voltage of 15 kV was used for imaging in the secondary electron scanning mode.

3D Electron Diffraction and High-Resolution Transmission Electron Microscopy (HRTEM). Thoroughly ground B-SSZ-43 powder (synthesized from OSDA Molecule 3, Figure 1) was dispersed in ethanol followed by an ultrasonic bath treatment for 5 minutes. A drop of the suspension was applied to a lacey carbon grid (Cu150P from Okenshoji Co., Ltd) and dried in air for 5 minutes. HRTEM images were acquired on a JEOL JEM 2100F microscope operated at 200 kV and equipped with a Gatan Ultrascan 1000 camera. Three-dimensional electron diffraction (3D ED) was conducted on a JEOL JEM-2100 microscope with LaB_6 cathode operating at 200 kV and equipped with a hybrid Timepix camera. Continuous rotation electron diffraction (cRED) data acquisition was controlled using the software *Instamatic* [6]. Twenty-nine individual datasets were collected from different crystals. In a typical experiment, a crystal was continuously rotated while ED frames are collected over a tilt range of ±60° with a rotation speed 0.45° s^{-1}. The exposure time was set up to be 0.5 s. The collected frames were processed with *XDS* software [7] for spot-finding, indexing, space-group assignment, data integration, scaling, and refinement. The structure was solved by using *Olex2* software [8] with the *SHELXT* dual-space algorithm [9] and further refined by *SHELXL* [10] with electron atomic structure factors [11].

Faulting simulation. DIFFaX software [12] was utilized to perform simulations of the faulted SSZ-43 structure. A three-layer model was used to simulate the PXRD patterns with faulting probability, α varied from 0 to 1. The DIFFaX simulations were performed by employing a Pseudo-Voight broadening profile and assuming that the layer width and number of stacked layers are infinite. PXRD patterns were simulated in the 2θ range between 2° and 30°, with a step size of 0.005°.

3. Molecular Modeling

The molecular modeling procedure was designed and programmed in the Pipeline Pilot platform [13] to facilitate high-throughput screening of OSDAs within the target zeolite structures. The van der Waals (vdW) interaction energy between the OSDA guest molecules and zeolite host framework was calculated by molecular mechanics simulations using Materials Studio software [14]. The CVFF force field [15] was selected for optimizing both the OSDA species and zeolite frameworks, and the most stable locations for the OSDAs were obtained by simulated annealing. All zeolite framework compositions used in the calculation were comprised of pure SiO_2 because the precise locations of heteroatoms within each framework are not known. We limited the modeling to vdW interaction energies between OSDA molecules and the target zeolite framework and did not include other inorganic cations or H_2O molecules in the calculation. The unit cell of zeolite framework structure was fixed during the calculation. A supercell was created when the OSDA molecular dimensions exceeded the zeolite unit cell dimensions. The geometry of the OSDA molecules were first optimized by using the CVFF force field and then allowed to relax after they were placed inside of the zeolite frameworks. Initially, one OSDA molecule was occluded per unit cell, and then the loading was increased until the minimum vdW interaction energy was reached.

4. Tables

Table S1. Atomic coordinates of the SSZ-43 polytype A structure (orthorhombic).

Atom	*x*	*y*	*z*
T1	0.7887(6)	0.2510(3)	0.6588(5)
T2	0.7084(4)	0.9982(2)	0.4186(3)
T3	0.2091(5)	0.6562(3)	0.5807(3)
T4	0.4607(5)	0.7538(3)	0.6601(4)
T5	0.1463(4)	0.8453(3)	0.5975(4)
T6	0.4651(4)	0.0627(2)	0.6580(3)
T7	0.4638(4)	0.6560(3)	0.5860(3)
T8	0.8671(4)	0.4340(3)	0.3410(3)
T9	0.4691(4)	0.3460(3)	0.5909(3)
T10	0.0381(4)	0.4964(2)	0.4127(3)
T11	0.6628(4)	0.0944(3)	0.3406(4)
T12	0.6695(3)	0.0059(3)	0.5997(3)
T13	0.2865(4)	0.0936(3)	0.3422(3)
T14	0.9648(4)	0.9073(2)	0.6576(3)
O1	0.8574(10)	0.2930(5)	0.6308(10)
O2	0.1759(17)	0.7443(11)	0.75
O3	0.6731(9)	0.9975(7)	0.5078(6)
O4	0.6667(9)	0.0477(6)	0.3835(11)
O5	0.8338(5)	0.9882(5)	0.4075(8)
O6	0.6447(7)	0.9546(6)	0.3783(8)
O7	0.1663(11)	0.6513(8)	0.4929(9)
O8	0.8482(10)	0.1184(6)	0.6289(10)
O9	0.6616(7)	0.1497(5)	0.5833(8)
O10	0.5287(10)	0.7937(6)	0.6216(8)
O11	0.5040(2)	0.2497(12)	0.75
O12	0.5007(9)	0.2062(4)	0.6232(7)
O13	0.2326(8)	0.8760(6)	0.6402(8)
O14	0.9682(7)	0.3686(6)	0.6097(7)
O15	0.5215(9)	0.6165(5)	0.6402(7)
O16	0.5377(19)	0.5546(9)	0.75
O17	0.5654(8)	0.0333(5)	0.6217(7)
O18	0.5158(10)	0.6517(6)	0.5006(6)
O19	0.7739(8)	0.4699(6)	0.3713(9)
O20	0.9754(9)	0.4532(6)	0.3729(8)
O21	0.1290(2)	0.9342(11)	0.25
O22	0.1780(12)	0.7931(6)	0.3834(10)
O23	0.3348(8)	0.7375(6)	0.3528(8)
O24	0.5524(8)	0.3813(6)	0.6297(10)
O25	0.3489(7)	0.3687(5)	0.6098(6)
O26	0	0	0.5
O27	0.0097(10)	0.5418(6)	0.3670(10)
O28	0.6520(2)	0.0875(13)	0.25
O29	0.3021(12)	0.1043(8)	0.25
O30	0.6623(8)	0.5416(7)	0.3561(8)
O31	0.8402(6)	0.9038(5)	0.6379(7)
O32	0.0185(12)	0.3987(8)	0.75

Table S2. Atomic coordinates of the SSZ-43 polytype B structure (monoclinic).

Atom	*x*	*y*	*z*
T1	0.2272(8)	0.3400(6)	0.8139(7)
T2	0.9762(8)	0.1589(6)	0.8114(7)
T3	0.4001(8)	0.6605(6)	0.1914(7)
T4	0.5732(8)	0.3407(5)	0.1208(7)
T5	0.6300(8)	0.5920(6)	0.3045(7)
T6	0.1745(8)	0.3409(6)	0.1266(7)
T7	0.3813(9)	0.1606(7)	0.5073(9)
T8	0.7544(8)	0.0955(6)	0.6924(7)
T9	0.7079(8)	0.4179(6)	0.0039(7)
T10	0.0365(8)	0.5868(6)	0.9933(7)
T11	0.3080(9)	0.4188(6)	0.3124(7)
T12	0.3390(8)	0.4052(6)	0.0125(7)
T13	0.5623(9)	0.4169(6)	0.3083(7)
T14	0.6242(8)	0.1613(6)	0.5065(7)
O1	0.8394(12)	0.4099(16)	0.0194(17)
O2	0.2660(16)	0.3944(14)	0.7392(15)
O3	0.6762(15)	0.3757(14)	0.0906(14)
O4	0.3652(17)	0.6174(14)	0.0926(12)
O5	0.8838(13)	0.3891(15)	0.7355(15)
O6	0.4608(13)	0.3816(15)	0.0663(16)
O7	0.6882(16)	0.6371(14)	0.0881(12)
O8	0.6269(17)	0.1145(14)	0.4122(12)
O9	0.0512(13)	0.1286(14)	0.0919(14)
O10	0.3150(16)	0.8606(14)	0.2496(15)
O11	0.6067(18)	0.9979(9)	0.3045(17)
O12	0.2257(17)	0.1343(14)	0.2364(12)
O13	0.5970(17)	0.1347(14)	0.2314(12)
O14	0.2526(16)	0.3761(14)	0.0672(15)
O15	0.4319(12)	0.4173(15)	0.2988(17)
O16	0.9510(2)	0.25	0.8010(2)
O17	0.5075(11)	0.1411(14)	0.5204(17)
O18	0.6670(17)	0.8793(15)	0.4136(12)
O19	0.5690(2)	0.25	0.1110(2)
O20	0.3520(2)	0.25	0.5120(2)
O21	0.1010(12)	0.1407(14)	0.8087(17)
O22	0.6673(17)	0.5045(8)	0.0076(17)
O23	0.3057(17)	0.3795(14)	0.4113(12)
O24	0.1560(2)	0.25	0.1070(2)
O25	0	0.5	0
O26	0.6510(3)	0.25	0.4940(3)
O27	0.7267(15)	0.1242(15)	0.5836(12)
O28	0.2649(18)	0.9957(9)	0.3105(18)
O29	0.5226(13)	0.8598(14)	0.2516(16)
O30	0.3940(2)	0.75	0.1610(2)
O31	0.9638(17)	0.3680(14)	0.9129(12)
O32	0.2410(3)	0.25	0.7930(2)

5. Figures

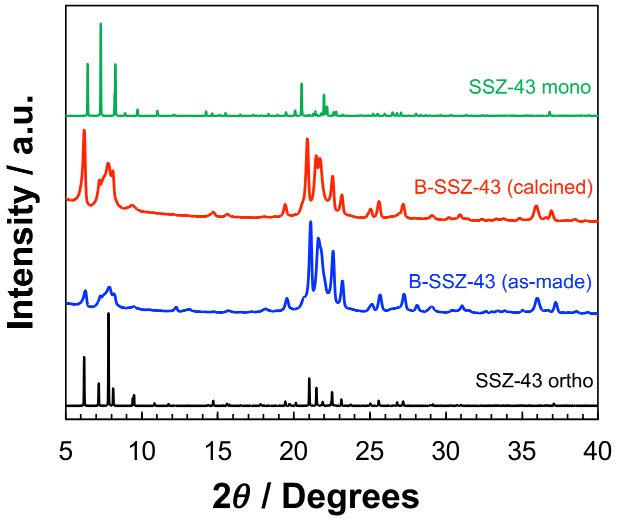

Figure S1. PXRD patterns for as-made B-SSZ-43 (blue line) prepared from Molecule 3, B-SSZ-43 after calcination (red line) in 100 cm^3 min^{-1} (g zeolite)$^{-1}$ dry air at 853 K for 6 h in a muffle furnace, and calculated patterns for the orthorhombic polytype A (black line) and monoclinic polytype B (green line) structures of SSZ-43, respectively. Patterns are normalized to their respective maximum intensities and offset vertically for clarity.

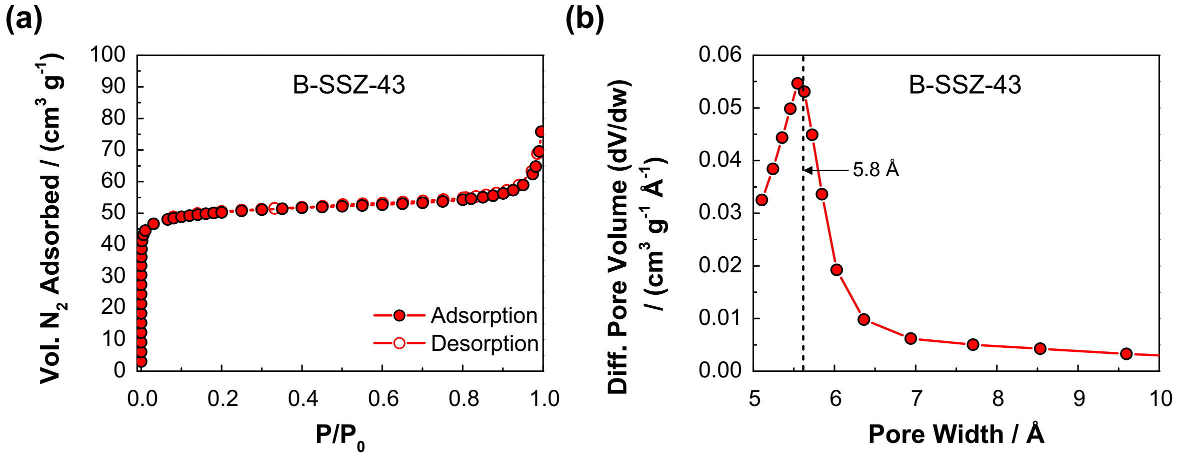

Figure S2. N_2 adsorption (77 K) for calcined B-SSZ-43 prepared from Molecule 3: (*a*) adsorption (closed circles) and desorption (open circles) isotherms, and (*b*) median micropore width based on cylindrical pore model (Saito-Foley method).

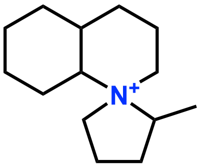

(7)

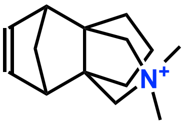

(8)

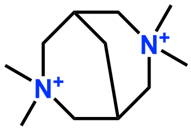

(9)

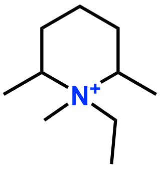

(10)

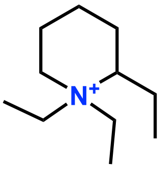

(11)

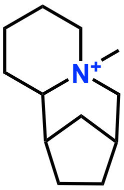

(12)

Figure S3. OSDA molecules 7-12 known to form competing ***STO** and **STF** phases.[2, 16-18]

(a)

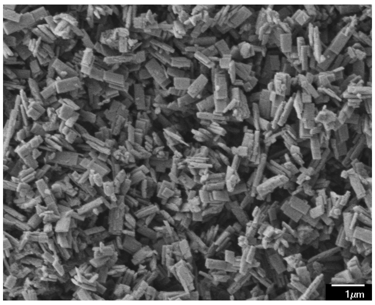

(b)

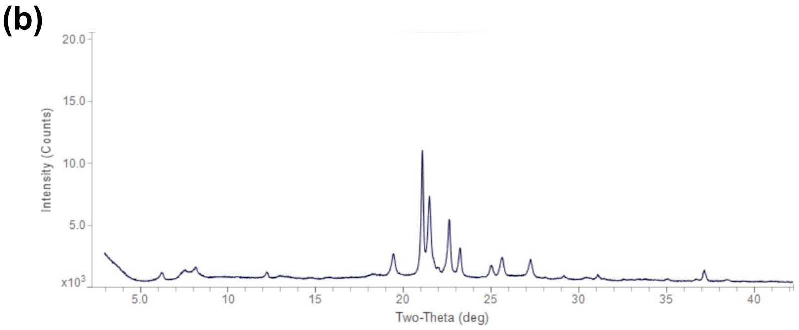

Figure S4. Al-SSZ-43 zeolite synthesized from OSDA Molecule A: (*a*) representative SEM image showing the plate-like morphology of the crystallites, and (*b*) PXRD patten of as-made material.

6. Accession Codes

CCDC 2091988 and 2091989 contain the supplementary crystallographic data for this paper. These data can be obtained free of charge via www.ccdc.cam.ac.uk/data_request/cif, or by emailing data_request@ccdc.cam.ac.uk, or by contacting The Cambridge Crystallographic Data Centre, 12 Union Road, Cambridge CB2 1EZ, UK; fax: +44 1223 336033.

7. References

[1] Shvets, O. V.; Zukal, A.; Kasian, N.; Žilková, N.; Čejka, A. The Role of Crystallization Parameters for the Synthesis of Germanosilicate with UTL Topology. *Chem. Eur. J.* **2008**, *14,* 10134-10140.

[2] Lee, G. S.; Nakagawa, Y.; Reynolds, R. N. Zeolite SSZ-43. *US Patent 5965104*, **1999**.

[3] Senapati, S.; Zimdars, J.; Ren, J.; Koller, H. Post-synthetic modifications of as-made zeolite frameworks near the structure-directing agents. *J. Mater. Chem. A.* **2014**, *2,* 10470-10484.

[4] Xie, D. Synthesis of molecular sieve SSZ-43. *US Patent 10384949*, **2019**.

[5] Petříček, V.; Dušek, M.; Palatinus, L. Crystallographic Computing System JANA2006: General Features. *Z. Kristallogr. Cryst. Mater.* **2014**, *229* (5), 345-352.

[6] Smeets, S. *Instamatic. https://Github.Com/Stefsmeets/Instamatic.* **2018**.

[7] Kabsch, W. XDS. *Acta Cryst. D.* **2010**, *66*, 125-132.

[8] Dolomanov, O. V.; Bourhis, L. J.; Gildea, R. J.; Howard, J. A. K.; Puschmann, H. OLEX2: a complete structure solution, refinement and analysis program. *J. Appl. Cryst.* **2009**, *42*, 339-341.

[9] Sheldrick, G. M. SHELXT - Integrated space-group and crystal-structure determination. *Acta Cryst. A.* **2015**, *71*, 3-8.

[10] Sheldrick, G. M. A short history of SHELX. *Acta Cryst. A.* **2008**, *64*, 112-122.

[11] Doyle, P. A.; Turner, P. S. Relativistic Hartree-Fock X-ray and electron scattering factors. *Acta Cryst. A* **1968**, *24*, 390-397.

[12] Treacy, M. M. J.; Newsam, J. M.; Deem, M. W. A General Recursion Method for Calculating Diffracted Intensities from Crystals Containing Planar Faults. *Proc. R. Soc. Lond. A* **1991**, *433*, 499–520.

[13] Pipeline Pilot, Accelrys, Inc.: San Diego, CA, **2013**.

[14] Materials Studio 6.0, Accelrys, Inc.: San Diego, CA, **2012**.

[15] Dauber-Osguthorpe, P.; Roberts, V. A.; Osguthorpe, D. J.; Wolff, J.; Genest, M.; Hagler, A. T. Structure and energetics of ligand binding to proteins: Escherichia coli dihydrofolate reductase-trimethoprim, a drug-receptor system. *Proteins*, **1988**, *4*, 31-47.

[16] Lee, G. S.; Nakagawa, Y.; Hwang, S. J.; Davis, M. E.; Wagner, P.; Beck, L.; Zones, S. I. Organocations in Zeolite Synthesis: Fused Bicyclo [l.m.0] Cations and the Discovery of Zeolite SSZ-48. *J. Am. Chem. Soc.* **2002**, *124*, 7024-7034.

[17] Nakagawa, Y.; Lee, G. S; Harris, T. V.; Yuen, L. T.; Zones, S. I. Guest/host relationships in zeolite synthesis: ring-substituted piperidines and the remarkable adamantane mimicry by 1-azonio spiro [5.5] undecanes. *Microporous Mesoporous Mat.* **1998**, *22*, 69-85.

[18] Wagner, P.; Nakagawa, Y.; Lee, G. S.; Davis, M. E.; Elomari, S.; Medrud, R. C.; Zones, S. I. Guest/Host Relationships in the Synthesis of the Novel Cage-Based Zeolites SSZ-35, SSZ-36, and SSZ-39. *J. Am. Chem. Soc.* **2000**, *122*, 263-273.
